# An alternative hypothesis testing strategy for secondary phenotype data in case-control genetic association studies

**DOI:** 10.3389/fgene.2014.00188

**Published:** 2014-07-01

**Authors:** Sharon M. Lutz, John E. Hokanson, Christoph Lange

**Affiliations:** ^1^Department of Biostatistics, University of ColoradoAurora, CO, USA; ^2^Department of Epidemiology, University of ColoradoAurora, CO, USA; ^3^Department of Biostatistics, Harvard School of Public HealthBoston, MA, USA; ^4^Channing Laboratory, Harvard Medical SchoolBoston, MA, USA; ^5^Institute for Genomic Mathematics, University of BonnBonn, Germany; ^6^German Center for Neurodegenerative Diseases (DZNE)Bonn, Germany

**Keywords:** secondary phenotype, case-control study, ascertainment, genetic association, proportional odds logistic regression

## Abstract

Motivated by the challenges associated with accounting for the ascertainment when analyzing secondary phenotypes that are correlated with case-control status, Lin and Zeng have proposed a method that properly reflects the case-control sampling (Lin and Zeng, 2009). The Lin and Zeng method has the advantage of accurately estimating effect sizes for secondary phenotypes that are normally distributed or dichotomous. This method can be computationally intensive in practice under the null hypothesis when the likelihood surface that needs to be maximized can be relatively flat. We propose an extension of the Lin and Zeng method for hypothesis testing that uses proportional odds logistic regression to circumvent these computational issues. Through simulation studies, we compare the power and type-1 error rate of our method to standard approaches and Lin and Zeng's approach.

## Introduction

For the analysis of secondary phenotype data collected in a case-control study, Lin and Zeng have proposed a method that properly reflects the case-control sampling (Lin and Zeng, 2009). This work is motivated by the challenges associated with accounting for the ascertainment when analyzing secondary phenotypes that are correlated with case-control status. Several methods have been proposed that accurately estimate the odds ratio of genetic variants for binary secondary phenotypes associated with case-control status, but most of these methods do not readily accommodate continuous secondary phenotypes (Greenland, 2003; Kraft, 2007; Richardson et al., 2007; Monsees et al., 2009; Li et al., 2010; Wang and Shete, 2011a,b; He et al., 2012; Li and Gail, 2012). While two of these methods use an inverse probability weighted (IPW) regression approach that can accommodate continuous secondary phenotypes, these methods focus on correcting for the bias in the estimator due to the ascertainment conditions and involve a known disease rate (Richardson et al., 2007; Monsees et al., 2009). Since this paper focuses on hypothesis testing versus estimation of disease-association parameters with an equal number of cases and controls, we do not present these methods here.

Alternatively, the Lin and Zeng method has the advantage of accurately estimating effect sizes for secondary phenotypes that are normally distributed or dichotomous (Lin and Zeng, 2009). Under the null hypothesis when the likelihood surface that needs to be maximized can be relatively flat, this method can be computationally intensive in practice. To circumvent these computational issues, we propose an extension of the Lin and Zeng method for hypothesis testing that uses proportional odds logistic regression. Since the approach by Lin and Zeng has the advantage that effect sizes can also be estimated, we recommend the following work-flow for the analysis of continuous secondary phenotypes.

1. Test all SNPs with our approach using proportional odds logistic regression since the vast majority of SNPs will be under the null hypothesis.
2. For the significant SNPs, apply Lin and Zeng's method to obtain parameter estimates and confidence intervals.

This proposed approach circumvents the computational issues encountered in the Lin and Zeng approach under the null hypothesis, but utilizes the Lin and Zeng's method to accurately estimate effect sizes for significant SNPs found in Step 1. Through simulation studies, we compare the power and type-1 error rate of our method to standard approaches and Lin and Zeng's approach.

## Methods

When the secondary phenotype is normally distributed, Lin and Zeng propose an adjusted score test that incorporates genetic associations with affection status into the test statistic and models the likelihood function as follows (Lin and Zeng, 2009):

(1) $$\begin{array}{l} \prod_{i\;=\;1}^{n}P(Y_{i},X_{i}|D_{i})=\prod_{i\;=\;1}^{n}{\left\{\frac{P(D_{i}=1|X_{i},Y_{i})P(Y_{i}|X_{i})P(X_{i})}{P(D_{i}=1)}\right\}}^{D_{i}}\\ \;{\left\{\frac{P(D_{i}=0|X_{i},Y_{i})P(Y_{i}|X_{i})P(X_{i})}{P(D_{i}=0)}\right\}}^{1-D_{i}} \end{array}$$

where *D* denotes the case-control status (1 = case and 0 = control), Y denotes the secondary phenotype, *n* denotes the total number of subjects, and X denotes the genotype of interest.

Lin and Zeng calculate $P(D_{i}=1)=\sum_{y}\sum_{x}P(D_{i}=1|x,y)P(y|x)P(x)$. The probability *P*(*D*|*X*, *Y*) is defined as a logistic regression model. They model *P*(*Y*|*X*) as a logistic regression for dichotomous Y or a linear regression for normally distributed Y. They maximize the likelihood with respect to *P*(*X*) by the Newton Raphson algorithm. In this framework, likelihood based statistics (i.e., Wald, score, and likelihood-ratio statistics) can be used to make inference.

The Lin and Zeng approach requires the secondary phenotype to be normally distributed and the method can be problematic under the null hypothesis since the likelihood surface that needs to be maximized can be relatively flat. Since Lin and Zeng's method estimates the parameters in the model by maximizing the likelihood given in Equation (1), the approach is numerically exhaustive when testing a large number of SNPs where a majority of the SNPs are under the null hypothesis. This is a result of the maximization of the likelihood function being difficult under the null hypothesis, since the surface can be flat due to the ascertainment condition.

If the primary goal of the secondary phenotype analysis is hypothesis testing as opposed to estimation of disease-association parameters, an alternative approach is to use the following likelihood composition, which ultimately does not require maximizing a relatively flat likelihood surface. Therefore, for the association testing of secondary phenotypes in case-control studies, we propose using a simpler break down of the likelihood that requires few assumptions.

(2) $$\prod_{i\;=\;1}^{n}P(Y_{i},X_{i}|D_{i})=\prod_{i\;=\;1}^{n}P(X_{i}|Y_{i},D_{i})P(Y_{i}|D_{i})$$

Under the null hypothesis, X is independent of Y given D and any confounders. The likelihood ratio test becomes

(3) $$\begin{array}{l} LRT=-2ln\left(\frac{\prod_{i\;=\;1}^{n}P(X_{i}|D_{i})P(Y_{i}|D_{i})}{\prod_{i\;=\;1}^{n}P(X_{i}|Y_{i},D_{i})P(Y_{i}|D_{i})}\right)\\ \;=-2ln\left(\frac{\prod_{i\;=\;1}^{n}P(X_{i}|D_{i})}{\prod_{i\;=\;1}^{n}P(X_{i}|Y_{i}D_{i})}\right)\sim \chi_{1df}^{2} \end{array}$$

As a result, one only needs to model *P*(*X*|*D*) and *P*(*X*|*Y*, *D*). For an additive genetic model, i.e., *X* = 0, 1, 2, corresponding to allele counts, instead of modeling the likelihood function, one can use a cumulative logistic regression model with proportional odds proportional for *P*(*X*|*D*) and the *P*(*X*|*Y*, *D*) such that

(4) $$\begin{array}{l} logit[P(X\leq j|Y,D)]=\alpha_{1j}+\delta_{1Y}Y+\delta_{1D}D\\ \;logit[P(X\leq j|D)]=\alpha_{0j}+\delta_{0D}D \end{array}$$

for *j* = 0, 1. To control for any known confounders, these covariates can be added to Equation (4). This model assumes the same effect for different cumulative logits (Agresti, 2002). If assumptions are not met then we recommend a link function for which the response curve is non-symmetric or adding a dispersion parameter. For imputed dosages, *j* becomes the number of dosage levels minus one, meaning the levels of X in the cumulative logistic regression are increased to the number of dosage levels minus one.

## Simulations

To assess the performance of this approach and compare it to Lin and Zeng's method, we conducted simulation studies following Lin and Zeng's manuscript with a MAF of 0.3, an additive mode of inheritance, and α = 0.01 level of significance (Lin and Zeng, 2009). We also compared both of these methods to the standard case-only method, control only method and combined case and control method where both cases and controls are included in the analysis. For the model of the secondary quantitative trait *Y* and the disease *D*,

(5) $$Y|X\;\sim N\left(\beta_{0}+\beta_{1}X,\sigma^{2}\right)$$

(6) $$P(D=1|X,Y)=\frac{exp\left(\gamma_{0}+\gamma_{1}X+\gamma_{2}Y\right)}{1+exp\left(\gamma_{0}+\gamma_{1}X+\gamma_{2}Y\right)}$$

where β_0_ = σ^2^ = 1, β_1_ = 0 under the null hypothesis and β_1_ = −0.12 under the alternative hypothesis. We let γ_2_ = *log*(2), γ_1_ varies from 0 to *log*(1.5), and γ_0_ was chosen such that the disease rate is 1% or 5%. For each combination of simulation parameters, we generated 1000 data sets with 500 cases and 500 controls.

**Figure 1:**
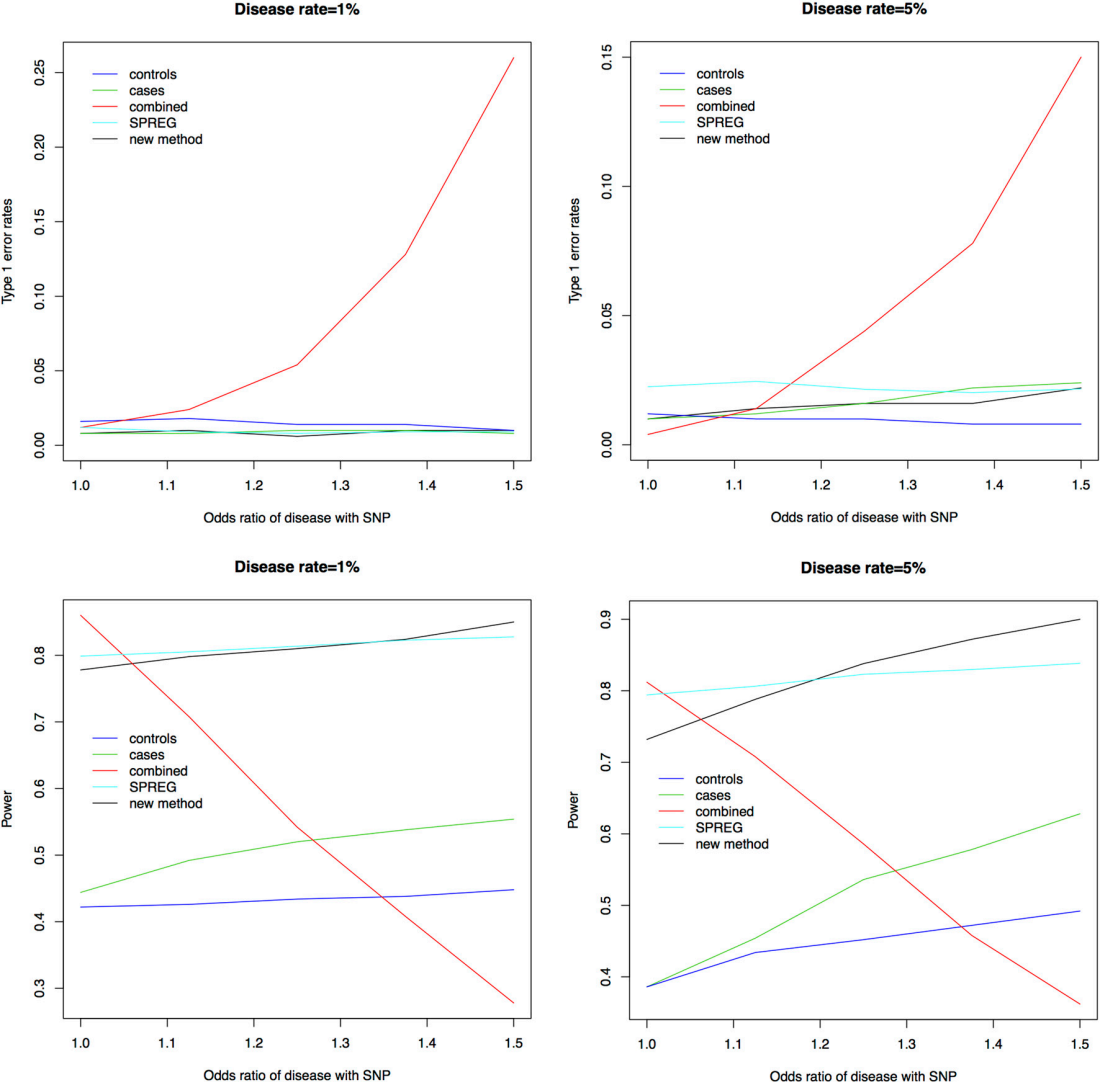
**Type 1 error rates and power for a disease rate of 1% and 5%**. As seen in the plots above the new method using proportional odds logistic regression maintains the type 1 error rate. The new method has similar power compared to Lin and Zeng's method called SPREG and superior power compared to the other methods.

**Figure 2:**
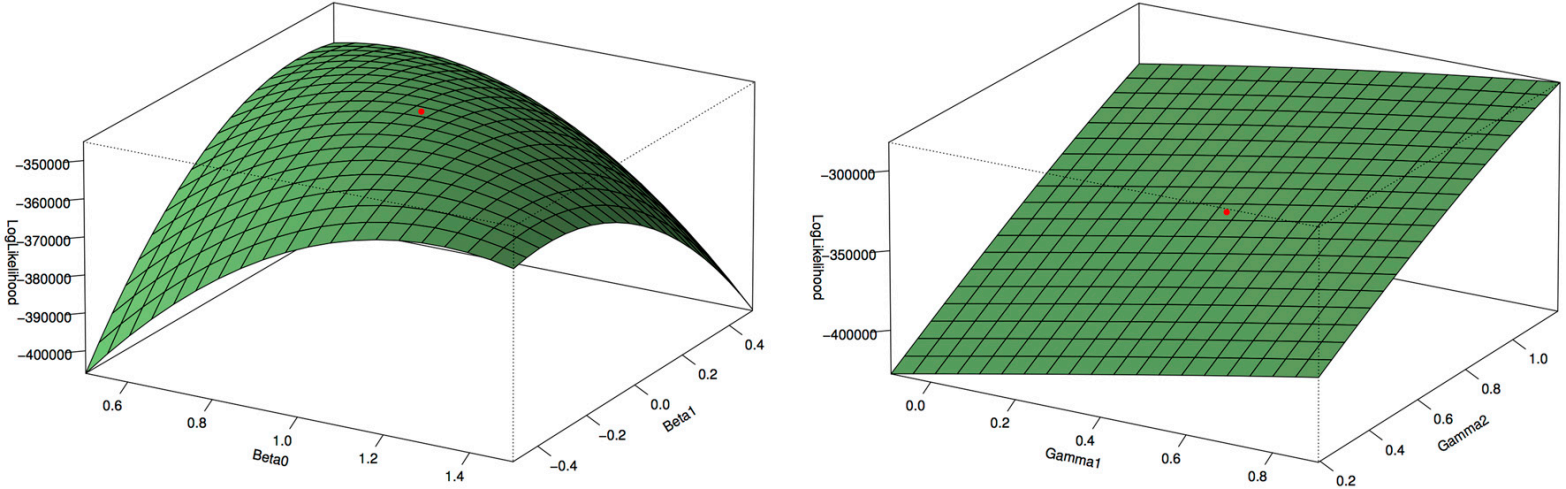
**Log Likelihood surface specified by Lin and Zeng**. The plot on the left is the log Likelihood specified by Lin and Zeng for varying values of β_0_ and β_1_ with all other parameters fixed at their true values and for data generated under the null hypothesis with γ_1_ = *log*(1.5) and the disease rate equal 5%. The plot on the right is the log Likelihood specified by Lin and Zeng for varying values of γ_1_ and γ_2_ with all other parameters fixed at their true values and for data generated under the null hypothesis with γ_1_ = *log*(1.5) and the disease rate equal 5%. The red dots on the plots represent the true maximum. The surface for β_0_ and β_1_ has a clear maximum whereas the surface for γ_1_ and γ_0_ is relatively flat, demonstrating the difficulty in maximizing the likelihood surface defined by Lin and Zeng under the null hypothesis.

Figure 1 shows the type 1 error rates and power for a disease rate of 1% and 5%. Our method, using the proportional odds logistic regression, maintains the type 1 error rate and has slightly higher power as compared to Lin and Zeng's method and superior power compared to the other methods. While the proposed method and Lin and Zeng's method have similar power, the proposed method is computationally more feasible under the null hypothesis than Lin and Zeng's method since it does not involve maximizing a relatively flat likelihood surface. The computing time for the proposed approach is under 1 s per SNP where as the software associated with the Lin and Zeng approach needs to be run multiple times if there are issues with convergence which can take 5 min to an hour per SNP. When running a GWAS with about 500,000 SNPs, this difference in computing time per SNP can be substantial. To examine this concept further, the plot on the left in Figure 2 shows the log Likelihood specified by Lin and Zeng for varying values of β_0_ and β_1_ with all other parameters fixed at their true values and for data generated under the null hypothesis with γ_1_ = *log*(1.5) and the disease rate equal 5%. The plot on the right is the log Likelihood specified by Lin and Zeng for varying values of γ_1_ and γ_2_ with all other parameters fixed at their true values, and for data generated under the null hypothesis with γ_1_ = *log*(1.5) and the disease rate equals 5%. The red dots on the plots represent the true maximum. The surface for β_0_ and β_1_ has a clear maximum whereas the surface for γ_1_ and γ_0_ is relatively flat, demonstrating the difficulty in maximizing the likelihood surface defined by Lin and Zeng under the null hypothesis.

## Discussion

While the power of the proposed method is comparable to the method of Lin and Zeng, the proposed approach does not have the issue of maximizing a flat likelihood surface under the hull hypothesis that can be computationally intensive. Since the proposed approach is limited in it's ability to accurately estimate effect sizes while the approach by Lin and Zeng has the advantage that effect sizes can be accurately estimated, we recommend the following work-flow for the analysis of secondary phenotypes.

1. Test all SNPs with the proposed approach using proportional odds logistic regression since the vast majority of SNPs will be under the null hypothesis.
2. For the significant SNPs, apply Lin and Zeng's method to obtain parameter estimates and confidence intervals.

By using our approach to test all the SNPs in the GWAS, the hypothesis testing can be done quickly and efficiently since our approach does not suffer from this issue of maximizing a flat likelihood surface under the null hypothesis. By obtaining parameter estimates for only the significant SNPs with Lin and Zeng's method, one can make sure that the likelihood is properly maximized which is too computational exhaustive to apply to the entire GWAS.

There are potential limitations associated with this strategy of combining two methodological approaches to reduce the computational burden while still being able to estimate the parameters of interest. While the two approaches have comparable power, a relatively small number of SNPs that are significant from the new approach may not be significant in the Lin and Zeng's method and vice versa. Also both approaches may have issues if the case control status is extremely correlated with the secondary phenotype. In this case, the secondary phenotype is not providing new information compared to the case-control status and these methods for testing secondary phenotypes in case-control genetic association studies are not applicable.

### Conflict of interest statement

The authors declare that the research was conducted in the absence of any commercial or financial relationships that could be construed as a potential conflict of interest.
